# Gamma Interferon: From Antimicrobial Activity to Immune Regulation

**DOI:** 10.3389/fimmu.2014.00667

**Published:** 2015-01-05

**Authors:** Howard M. Johnson

**Affiliations:** ^1^Microbiology and Cell Science, University of Florida, Gainesville, FL, USA

**Keywords:** interferon, antiviral, SOCS, JAK/STAT pathway, pathogens, cell signaling

Gamma interferon (IFNγ) was discovered in 1965 as an antiviral activity in white blood cell cultures stimulated with the lymphocyte mitogen phytohemagglutinin (1). At that time, IFNγ, as was the case with the much earlier discovered type I IFNs, was thought to strictly function as an antiviral. It was noted, however, that IFNγ was more sensitive than type I IFNs to extremes of pH and temperatures of 56°C or higher, a suggestion that it was a different kind of IFN.

We played a key role in the first demonstration that IFNγ along with the type I IFNs regulated immune functions (2–4). These discoveries were confirmed and extended by others, making IFNs the first well-characterized cytokines. The reviews presented here provide important insight into our current understanding of the structural basis of the mechanism of specific gene activation by IFNγ, how its activity is regulated by immune modulators called suppressors of cytokine signaling (SOCS) and regulatory T cells, its effector role in autoimmunity, and how it functions as a key factor in host defense against viral and parasitic diseases.

The first three articles deal, respectively, with a non-canonical model of IFNγ signaling (5), the role of IFNγ receptor in such signaling (6), as well as epigenetic aspects of activation of the IFNγ gene (7). SOCS and regulatory T cells (Tregs) play the key role as governors of signaling by cytokines such as IFNγ. Too much regulation can be detrimental in immune regulation of diseases such as cancer while too little can result in autoimmune disease. Thus, the fourth article addresses cross-talk between SOCS1 and Tregs in the context of regulation of IFNγ (8). The cross-talk between the systems is a novel observation with SOCS1 playing the hierarchical role.

With respect to viral diseases, IFNγ is discussed in the context of human immunodeficiency virus-1 (HIV-1) (9) and herpes simplex virus-1 (HSV-1) (10). These are difficult viruses to control in an infectious setting. HSV-1 attacks the immune system as well as the central nervous system (CNS). The HIV-1 review points out the complex relationship between IFNγ and this virus where both therapeutic as well as exacerbative effects on HIV-1 pathogenesis have been attributed to IFNγ. HSV-1 is a difficult, persistent neurotropic infectious virus. The relationship of IFNγ to HSV-1 pathogenesis is defined more clearly than is the case for HIV-1. For example, IFNγ plays a key role in preventing the virus from exiting the latency state.

IFNγ is a key player in regulation of protective immunity against blood-stage malaria, and this is discussed in the context of gamma/delta T cells (11). Specifically, certain gamma/delta T cells produce IFNγ after *Plasmodium* infection, which is shown to be involved in dendritic cell reduction of *Plasmodium* parasites in the blood. IFNγ is probably the key cytokine in host defense against *Leishmania* infections in general although different *Leishmania* species may respond differently against this cytokine (12). Even so, IFNγ appears to function cooperatively with chemokines such as CXCL10 in CD8^+^ T cell response to *Leishmania*-infected cells.

Finally, IFNγ as well as type I IFNs may function cooperatively in promotion of Sjogren’s syndrome as shown by upregulation of IFN response genes (IRGs) (13). Sjogren’s syndrome is characterized by chronic immune attacks against exocrine glands (such as salivary glands) leading to exocrine dysfunction. An SOCS1 mimetic plays an important role in inhibiting the inflammatory aspect of autoimmune diseases such as multiple sclerosis as pointed out in the fourth review (8). Thus, given the importance of the IFNs in Sjogren’s syndrome, it would seem that it would be a candidate for potential SOCS1 mimetic therapy based on the experience with other autoimmune diseases. The SOCS and Sjogren’s syndrome reviews as well as others presented here, therefore, are interrelated in terms of approaches to dealing with diseases where IFNγ may play either a positive or negative role.

## Conflict of Interest Statement

The author declares that the research was conducted in the absence of any commercial or financial relationships that could be construed as a potential conflict of interest.
